# Role of organizational network analyses to advance workforce inclusion and belonging: a scoping literature review

**DOI:** 10.3389/fpsyg.2025.1708522

**Published:** 2025-12-03

**Authors:** Patrick L. Decker-Tonnesen, Sherry S. Chesak, Laura E. Walker, Katharina Kohler, Sean Phelan, Marshall S. Gunnels, Kara L. Saliba, Anjali Bhagra

**Affiliations:** 1 Office of Belonging, Mayo Clinic, Rochester, MN, United States; 2 Division of Nursing Research, Mayo Clinic, Rochester, MN, United States; 3 Department of Emergency Medicine, Mayo Clinic, Rochester, MN, United States; 4 Department of Medicine, University of Cambridge, Cambridge, United Kingdom; 5 Health Care Delivery Research, Mayo Clinic, Rochester, MN, United States; 6 Nursing Administration, Mayo Clinic, Rochester, MN, United States; 7 Office of Belonging, Mayo Clinic, Phoenix, AZ, United States

**Keywords:** organizational network analysis (ONA), workplace inclusion, social networks, workplace belongingness, workplace dynamics

## Abstract

Organizational Network Analyses (ONAs) are tools used to explore the strengths and challenges in the human relationships that occur within organizations. ONAs consist of matrices of informal and formal connections that help to identify potential patterns of inclusion and exclusion across organizational systems. Understanding the directions of workplace relationships is critical for advancing workforce inclusion and belonging. To better understand the efficacy of organizational networks for inclusion and belonging, this scoping literature review included fifteen studies that met inclusion criteria and were placed into five categories including: Studies With Gender-Related Outcomes; Studies With Race, Ethnicity, and Culture-Related Outcomes; Studies With Cross Gender-Related and Race, Ethnicity, and Culture-Related Outcomes; Studies With Academic Medical Setting-Related Outcomes; and Studies With Miscellaneous Outcomes. Results demonstrated ONAs are a useful tool for organizations to better understand barriers to inclusion and belonging, including for employee gender and race. Additionally, ONAs help provide information to organizations on network differences and connectivity within groups. The findings of this review warrant future research to further assess how ONAs interact with dimensions of difference and workplace inclusion and belonging.

## 1 Introduction

Organizational networks are critical to understanding the relationships between employees within and across teams, levels, and departments. Organizational networks are defined as the relationships that exist within a group, team, department or organization as a whole (Cross et al., 2007). While such relationships and networks may vary between one organization and another, common relational trends and patterns tend to exist (Bergenholtz and Waldstrøm, 2011). Understanding the directions and nature of these trends, along with the benefits and opportunities they present, is imperative in advancing workforce inclusion and belonging.

Network relationships in organizations demonstrate the prevalence and interconnection of hierarchy, symmetry, bidirectionality and collinearity (de Lurdes Veludo et al., 2006). Most organizations have established power structures in the form of hierarchies, wherein clear boundaries exist between employees (Zaleznik, 2022). Such structures influence the development of social networks within organizations and are rooted in traditional forms of power and control within society. For example, traditional structures of male-based hierarchies in society, or patriarchies, have existed for thousands of years and have been shown to continue to disadvantage women to this day in the workplace (Zhu and Chang, 2019). In one recent study, the review of traditional forms of hierarchies in organizations is responsible for increasing women’s benevolence with sexism in the workplace (Radke et al., 2018).

Historically, underrepresented populations, such as women, veterans, people of color, members of the lesbian, gay, bisexual, transgender, and queer (LGBTQ) communities, people with visible and invisible disabilities, and older adults are employees that face added barriers when establishing and maintaining relationships in the workplace (Dennissen et al., 2019, 2020; Shore et al., 2018; Sloan et al., 2013). Awareness of these network relationships is important as they play a crucial role in career advancement and can effect underrepresentation of the aforementioned populations in leadership positions (Singh et al., 2002). Furthermore, current research demonstrates that network relationships play an important part in the sense of inclusion and belonging experienced by underrepresented populations (Filstad et al., 2019). However, these relationships are often strongest within underrepresented groups and not well developed across other groups, (Trenerry and Paradies, 2012).

Organizations across the globe are investing more resources than ever before into workplace belonging (CISION PR Newswire, 2021). To understand the longitudinal effects of such efforts, organizational network analyses (ONAs) are useful tools that can expose the strengths and cracks in the foundations of workplace relationships. ONA is a methodology that maps informal and formal relationships to identify potential patterns of inclusion and exclusion within and across organizations.

ONA tools are particularly helpful in exploring the barriers that exist for underrepresented populations and remote workers within organizations (Cross et al., 2021). For example, research demonstrates that women are often required not only to outperform their male colleagues in task completion, but also expected to constrain their emotions at work (Cross et al., 2021). This drives women to prioritize job duties in lieu of developing and nurturing professional relationships. This cycle may prevent women from forming the beneficial connections that are often the cornerstone of career advancement. Furthermore, ONA has demonstrated a tendency for homophily in gender networks in the workplace and, given the fact that the majority of senior leaders are men, women who largely network with other women tend to have limited interactions with senior colleagues and thus are placed at a disadvantage for promotion (Suurna and Leibbrandt, 2022).

Based on the literature, the research team set out to explore the efficacy and application of ONA for inclusion and belonging in the workplace and developed the following research question: what are the outcomes of studies that used ONA as a method to assess and/or influence inclusion and belonging in the workplace? Additionally, the research team was interested in examining the prominent research methods and categories related to workplace inclusion and belonging as studied in ONA.

### 1.1 Key terms

**Figure 1:**
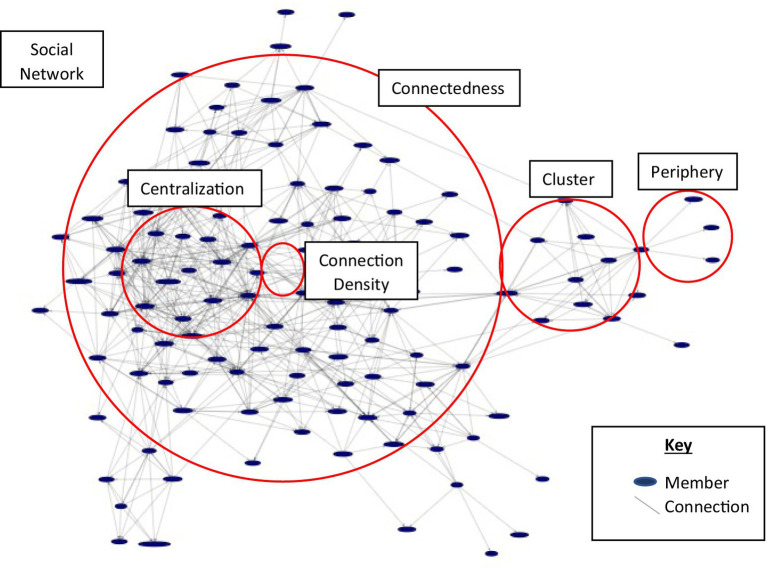
Organizational network example and key terms. Key includes member, which represented an individual person within a network and connection, which represented a relationship that existed between two people within a network. Definitions include Social network or social network analysis (SNA) is defined by the measuring of individuals who exist within a population (such as a group, organization, or community) and the relationships they hold with each other; Cluster or group is described as the closeknit and often interwoven relationships that occur within a population subgroup; Centralization or centrality is described by the degree to which groups display power dynamics, such as through hierarchies; Connection density is defined as the total number of connections that exist within a network out of the total possible number of connections; Connectedness described the overall proximity of relationships that exist within a group; Periphery is defined by the far ends or silos of a network that are farthest from the center; and Internal and external collaboration, which are defined by the presence of network relationships that promote network expansion and that exist within and outside of a network, respectively.

There are several key terms associated within organizational networks that will be defined for clarity throughout this paper and are included in the Figure 1. The first term is *social network* or *social network analysis (SNA)*, which is defined by the measuring of individuals who exist within a population (such as a group, organization, or community) and the relationships they hold with each other (Marion et al., 2003). Social network relationships are a distinct component of ONAs because they form the basis for understanding the patterns and trends of the relationships that exist within an organization. While like ONAs, social networks focus on specific relationships that exist between individuals within a group versus how relationships may be influenced within the context of a workplace (Marion et al., 2003). The second term is *cluster* or *group*, which describes the closeknit and often interwoven relationships that occur within a population subgroup (Wölfer and Hewstone, 2017). Clustering or grouping occurs when individuals within a network have a higher-than-random probability of being closely connected based on their relationship patterns (Wölfer and Hewstone, 2017).

The third term is *centralization* or *centrality* which describes the degree to which groups display power dynamics, such as through hierarchies (Wölfer and Hewstone, 2017). A network that has mostly equal density is more likely to have higher levels of reciprocity across its relationships. The fourth term is *connection density*, which refers to the total number of connections that exist within a network out of the total possible number of connections. Connetion density helps to identify why and where network relationships exist. The fifth term is *connectedness*, which refers to the overall proximity of relationships that exist within a group. Connectedness is useful for demonstrating how close or far apart members are from each other within a network. The sixth term is *periphery*, which describes the far ends or silos of a network that are farthest from the center. Members within a network that exist at the periphery may be least engaged as they are less likely to have as many connections as those near the center. The final terms are *internal* and *external* collaboration, which refer to the presence of network relationships that promote network expansion and that exist within and outside of a network, respectively. External and internal collaboration are important for undersatnding the opportunities for relationship expansion inside and outside of existing networks (Katerndahl, 2012).

The most accounted forms of networks include *organizational,* which primarily focus on the dyadic relationships that exists within and across teams and departments; *social*, which span the individual and collective social relationships of groups; *ego*, which include the unique relationships of each individual member in a group or on a team; and *ecomaps*, which focus on the proximity and intimacy of an individual’s relationship with other people and their social environment (de Lurdes Veludo et al., 2006).

## 2 Methods

The research team engaged in a scoping literature review and conducted a search of several databases from inception through August 31, 2022, limited to English language articles. The databases included Ovid MEDLINE(R) and Epub Ahead of Print, In-Process and Other Non-Indexed Citations and Daily 1946+, Ovid APA PsycInfo 1806+, Scopus via Elsevier (1788+), Web of Science Core Collection via Clarivate Analytics (1975+), Science Direct via Elsevier, and EBSCO MegaFILE (Business Source Premier, Academic Search Premier). Controlled vocabulary and keywords were used to search for studies describing organizational network analysis and belonging within organizations. A representative sample of search terms that were used include, but are not limited to, organizational network, social network, analysis, inclusion, connectedness, belonging, equity, workplace, corporate, industry, and organization in various combinations. A full description of the search methodology is available in the Appendix.

Inclusion criteria included: articles with the main topic containing validity and utility of ONA as a tool for connectedness within organizations, and/or ONA as a tool to measure trends of belonging and to help create action items to enhance belonging and/or, ONA as a tool to measure or impact inclusion within organizations. Exclusion criteria included: animal and lab studies, children as participants, studies not related to employees in a workplace setting, and those focused on “social network” in reference to social media networks.

**Figure 2:**
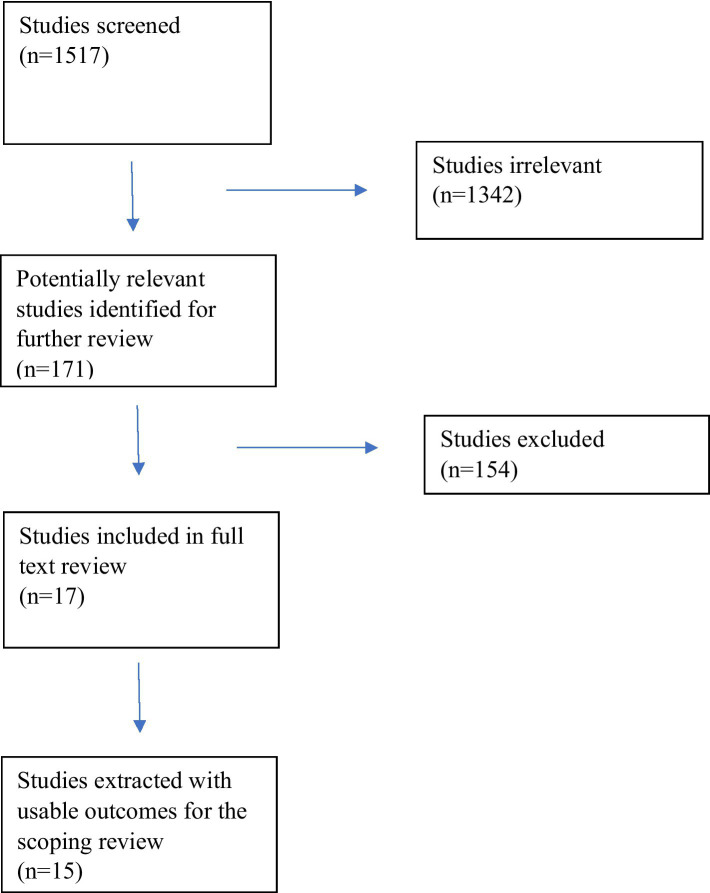
PRISMA diagram.

Seventeen manuscripts fit the inclusion criteria utilizing the Preferred Reporting Items for Systematic reviews and Meta-Analyses (PRISMA), and fifteen studies were extracted as including usable outcomes (Figure 2). The manuscripts were then categorized according to their foci in the following groupings: gender-focused outcomes (*n* = 6), race, ethnicity, and culture-related outcomes (*n* = 3), crossed both of the previous categories (*n* = 1), academic medical setting-related outcomes (*n* = 2), and miscellaneous (*n* = 3) (subjects in the 3 studies included: individuals with disabilities, employees of a multinational corporation, and LGBTQ employees in the workplace) (Table 1).

**Table 1:** Studies by categories of outcomes.

Title	Author	Published year	Journal	Study design	Number/types of participants	Strength of evidence
Studies with gender-related outcomes						
Mapping exclusion in the organization: organizational network analysis can reveal ways to bolster inclusivity	Carboni et al.	2022	MIT Sloan Management Review	Cohort	7,000 tech company employees	Strong
Mapping social exclusion in STEM to men’s implicit bias and women’s career costs	Cyr et al.	2021	Proceedings of the National Academy of Sciences of the United States of America	Survey	1,247 STEM professionals who work in teams	Strong
Do women in science form more diverse research networks than men? An analysis of Spanish biomedical scientist	Diaz-Faes et al.	2020	PLoS One	Survey	4,758 biomedical scientists	Strong
Cross-cultural connections: Leveraging social networks for women’s advancement	Hunt et al.	2009	The Glass Ceiling in the 21st century: understanding barriers to gender equality	Qualitative	5 women of diverse cultures	Weak
Connectedness is critical: a social network analysis to support emerging women leaders in global health	Lopez Hernandez et al.	2022	Preventative Medicine: An International Journal Devoted to Practice and Theory	Survey	114 women’s leadership program participants	Moderate
Shades of power: Network links with gender quotas and corporate governance codes	Mateos de Cabo et al.	2021	British Journal of Management	Longitudinal	71,300 directors of firms	Strong
Studies with race, ethnicity, and culture-related outcomes						
The Influence of Ethnic Diversity on Social Network Structure in a Common-Pool Resource System: Implications for Collaborative Management	Barnes-Mauthe et al.	2013	Ecology and Society	Cohort	159 fishers in Hawaii	Moderate
Outside of the corporate mainstream and excluded from the work community: A study of diversity, job satisfaction and well-being	Mor Barak et al.	2002	Community, Work, and Family	Survey	3,400 employees in the high-tech industry in Southern California	Strong
Structuring for team success: The interactive effects of network structure and cultural diversity on team potency and performance	Troster et al.	2014	Organizational Behavior and Human Decision Processes	Cohort	456 participants in an organizational analysis course at European business school	Moderate
Studies with cross gender-related and race, ethnicity, and culture-related outcomes						
The Application of Social Network Analysis as an Assessment Tool in the Field of Diversity and Inclusion	Garcia et al.	2008	Diversity Factor	Case report	Employees of a woman-owned boutique management consulting firm (*n* not specified)	Moderate
Studies with academic medical setting-related outcomes						
Evolution of the research collaboration network in a productive department	Katerndahl	2012	Journal of Evaluation in Clinical Practice	Cross-sectional	19–26 (yearly survey respondents) faculty in a Department of Family and Community Medicine	Moderate
Networking matters: a social network analysis of the association of program directors of internal medicine	Warm et al.	2018	Teaching and Learning in Medicine	Cohort	370 program directors in ACGME accredited internal medicine programs in the United States	Moderate
Studies with miscellaneous outcomes						
Out of sight and out of mind? Networking strategies for enhancing inclusion in multinational organizations	Farh et al.	2021	Journal of Applied Psychology	Cross- sectional	409 employees and leaders of a multinational corporation	Moderate
Social networks and career advancement of people with disabilities	Kulkarni	2012	Human Resource Development Review	Conceptual paper; scoping review	Individuals with disabilities (*n* not specified)	Moderate
Improving the wellbeing of LGBTQ+ employees: Do workplace diversity training and ally networks make a difference?	Perales et al.	2022	Preventative Medicine: An International Journal Devoted to Practice and Theory	Cross -sectional	31,277 employees from 149 Australian organizations	Strong

### 2.1 Study characteristics

The reviewed papers included a wide range of study designs, including cohort (*n* = 4), survey (*n* = 4), cross-sectional (*n* = 3), qualitative (*n* = 1), longitudinal (*n* = 1), case report (*n* = 1), and conceptual/scoping review (*n* = 1). The number of participants across studies varied widely with a range of 5–71,300; two studies did not specify the number of participants. Publication dates ranged from 2002 to 2022. Strength of evidence was considered based on relevance to the research question and included strong (*n* = 6), moderate (*n* = 8) and weak (*n* = 1). The Table outlines the study characteristics for each paper. Studies were categorized and will be described based on five main groups, including: Studies With Gender-Related Outcomes; Studies With Race, Ethnicity, and Culture-Related Outcomes; Studies With Cross Gender-Related and Race, Ethnicity, and Culture-Related Outcomes; Studies With Academic Medical Setting-Related Outcomes; and Studies With Miscellaneous Outcomes.

## 3 Findings

### 3.1 Studies with gender-focused outcomes

The largest cohort of manuscripts addressed gender-focused outcomes. The topics investigated, approaches, and outcomes were highly heterogeneous among the six papers included. The geographic areas spanned the U.S., Spain, Europe and global corporations with a presence in multiple countries. The hypotheses were equally varied and included the concept that women’s networks would be more diverse and have a more open structure with different brokerage roles compared to men (Díaz-Faes et al., 2020), that woman-to-woman networks are important for career growth and that ONA could be a beneficial tool to inform future interventions (Hernandez et al., 2022), that variability in social connections between and among men and women would result in different network characteristics and outcomes (Cyr et al., 2021), that voluntary versus mandated gender inclusivity on boards of directors would have varying results that could be seen in ONA (de Mateos Cabo et al., 2021), and finally that ONA would provide insights related to gender imbalances in hiring and promotion (Carboni et al., 2022). Additionally, a focus group was reported evaluating how networking, social identities and social capital affect connectivity to the organizational in-group (Hunt et al., 2009).

Most studies constructed an ego network and collected additional information such as demographics, position, assessments of personal connectivity, fit and engagement. Centrality was a common analysis indicating influence (Carboni et al., 2022), overall connectivity (Hernandez et al., 2022), and for comparison between genders (Cyr et al., 2021; de Mateos Cabo et al., 2021). Some authors assessed general network structure and presence of subnetworks (Carboni et al., 2022; Díaz-Faes et al., 2020; de Mateos Cabo et al., 2021).

The conclusions of the studies were supportive of the use of ONA to improve the understanding of how women and men function differently within organizations and supported the use of ONA tools to analyze initiatives aimed at improving belonging within the workplace. Recommendations included using active strategies to intentionally pull in more women who are at the edges of networks and connecting them with more critical teams, to restructure working groups for strategic relationship, and to use network intermediaries to improve collaboration. Network analysis and stratification by gender can elucidate how varying patterns of relationships play out in professional organizations and give an opportunity to use those relationships to improve wellbeing and inclusivity at work, as well as organizational performance.

The limitations present in these studies include that universally binary gender definitions were used across the studies. Response rates were low and at times there was incomplete data on gender. de Mateos Cabo et al. (2021) filled in missing responses using name/gender concordance and internet searches, which could introduce bias. Another interesting limitation from Cyr et al. (2021) was that it appears that those who have a higher status within an organization are less attentive to those they interact with and there was discordance compared to those who reported ties to them and the ties they reported. Varying areas of investigation, hypotheses, interventions, and outcomes makes generalizing the utility of ONA from these publications difficult; however, despite these variations all studies reported beneficial outcomes and significant insights when using ONA.

### 3.2 Studies with race, ethnicity, and culture-related outcomes

Three studies recognized the value and power of organizational networks for outcomes related to race, ethnicity, and culture. For example, one study involved the assessment of whether members of racial and ethnic minority employee groups were more likely to feel excluded, and the potential consequences of exclusion (Barak and Levin, 2002). Another study analyzed whether cultural differences amongst organizational network members of self-managed teams, as well as the connectedness of the team members, influenced the success of a team (Tröster et al., 2014). Finally, a third study examined the effect of ethnicity on network homophily, network structure, and cross-scale linkages within Hawaii’s longline fishery network (Barnes-Mauthe et al., 2013).

It was found that members of racial and ethnic minorities were more likely to feel excluded, contributing to decreased job satisfaction and a lower sense of well-being (Barak and Levin, 2002). Furthermore, a lack of ethnic differences within groups can result in the formation of discrete networks and result in a homophily effect that inhibits collaboration from group to group (Barnes-Mauthe et al., 2013). Conversely, highly cohesive organizational networks provide equal access to opportunities and resources for people who might otherwise be excluded or underrepresented, such as those who are members of minority groups. Network heterogeneity tends to lead to divergent viewpoints within a given network, which in turn potentially increases collaboration and network resiliency (Barnes-Mauthe et al., 2013). Connections that bridge ethnic and cultural ties may be few, but significant, in their impact on favorable outcomes (Barnes-Mauthe et al., 2013).

Various limitations were evident in attempts to glean information regarding organizational networks as it relates to race, ethnicity, and culture. Authors recognized that there is limited consensus on how observable identifiers such as nationality, gender, and race, may impact team performance. Theoretically, demographically diverse teams would leverage the mixed skills of the individuals, which would lead to positive group outcomes; however, there is evidence that dynamics within heterogenous groups may lead to interpersonal conflict and subsequent drop in team performance. Controlling for variables such as formal leadership, expertise, and prior performance, Tröster et al. (2014) studied 92 student teams during an eleven-week period European business course. Using descriptive statistics and correlations amongst variables, the authors concluded that team success (potency) is interdependent upon both the diversity of the team and the organizational network structure. As differences within a team increased, so did the positive influence of the organizational network upon the group’s capacity to achieve their goals. Demographically diverse teams appear to require greater network centrality to best facilitate top performance. However, the authors caution that excessive centralized influence could lead to disproportionate control and negatively influence team performance (Tröster et al., 2014).

### 3.3 Studies with cross gender-related and race, ethnicity, and culture-related outcomes

One study intersected two categories examining both gender-focused and race, ethnicity, culture-related outcomes. In this study, the authors describe ONA as a methodology that extends beyond simply measuring the number of diverse employees and instead focuses on understanding how diverse employees engage in decision-making and problem-solving together (Garcia et al., 2008). To further elucidate ONA efficacy in promoting inclusion and belonging, the authors describe the application of ONA using their own firm, Philosophy IB (PIB), as a case study. PIB is a woman-owned boutique management consulting firm, with 65% of its employees being first-generation Americans. Diversity is of paramount importance to PIB, with ethnic minorities representing over half the firm and women making up the majority of the firm’s partners (Garcia et al., 2008). The firm was planning an expansion, and management sought to utilize ONA to maintain the culture of diversity at PIB by exploring the extent to which working relationships were affected by race and gender (Garcia et al., 2008). Employee survey data was processed using network visualization and quantitative analysis techniques. The authors reported that at the organizational level, the data showed problem-solving relationships were not affected by race or gender; however, an additional sub-group analysis was performed to investigate at the individual level. Again, the data showed neither gender nor race affected problem-solving relationships to any statistically significant degree at the individual level. Mirroring the previous analysis, the individual level data showed neither gender nor race affected problem-solving relationships to any statistically significant degree. They interpreted the results to indicate that neither race nor gender affect how people work with each other at the company. Management planned to utilize ONA going forward to monitor the culture of diversity that PIB prides itself on.

### 3.4 Studies with academic medical setting-related outcomes

Two studies reported outcomes of trials that occurred in academic medical settings. The setting for the first study was a Department of Family and Community Medicine at an academic medical center (Katerndahl, 2012). The author aimed to identify how collaboration networks can impact research growth of a developing Family and Community Medicine department at the University of Texas Health Science Center at San Antonio over the course of 13 years (*n* = 17–26). Over this timeframe, the department implemented a plan to increase its scholarly activity, including policies, expectations, educational activities, and yearly evaluations. Faculty in the department were surveyed yearly from 1987 to 1999 and outcome measures included connectedness (density, mean degree, centralization and transitivity), distance (average distance and cohesion), groupings (E-I Index and clustering coefficient) and heterogeneity of distribution (core-periphery measure).

The patterns of network collaborations over the years were assessed using SNA and the findings indicated that research development occurred in three phases: (1) an initial phase of increasing centralization and collaboration in a single subject area; (2) a maintenance phase where there was high faculty turnover and cohesion and transitivity declined; and (3) an expansion phase in which there was stable department leadership and new researchers were actively recruited; trends observed during this phase included a decline in clustering coefficient and a steady rise in measures of centrality for external collaboration. The author concluded that the development phase relied heavily on a centralized network with external collaboration; within the maintenance phase connectedness and grouping varied, potentially due to faculty turnover; and in the expansion phase, there was a renewed importance of external collaboration.

In the second study, Warm et al. (2018) studied the strength of connections between 370 program directors from multiple organizations who were in the Association of Program Directors in Internal Medicine (APDIM) and its Education Innovations Project subgroup. They compared the associations between the strength of their connections and characteristics of program directors (participation in programs, age, gender, rank, location, burnout levels, desire to resign). SNA was used to measure out-degree centrality, in-degree centrality, and eigenvector centrality. Their findings indicated that the only factors that were associated with higher centrality were completion of the APDIM survey, being in a university-based program, Educational Innovations Project participation, and higher academic rank.

### 3.5 Studies with miscellaneous-related outcomes

Farh et al. (2021) examined feelings of inclusion and centrality as a source of advice in a multinational pharmaceutical corporation. They found that employees in headquarter sites had an advantage in feeling included and having professional advice ties over employees in subsidiary country sites. However, employees of subsidiary country sites experienced better inclusion and more professional advice ties when they collaborated on cross-country projects and tasks, especially if their team leader was well-connected. Based on their study, Farh et al. (2021) assert that inclusion and belonging in an organization requires feeling like one’s opinions are respected by the in-group, in this case, employees in headquarter country sites. They also conclude that well-connected leaders in the non-headquarter sites provide “social credentials” for lower-status site members’ advice to be considered valuable. The authors recommend that outgroup members may seek to become sources of advice as a way to be more included, and further suggest that organizations encourage cross-group collaborations and employ well-connected site leaders to foster that sense of inclusion.

In a conceptual paper, Kulkarni (2012) described how social network factors work to limit the career mobility of people with disabilities. The author argues that (1) people with disabilities may have strong ties with few like individuals but are less likely to have dense social networks. (2) People with disabilities are more likely to avoid or be excluded from social ties (e.g., friendships) with those they have professional ties with. (3) People with disabilities are less likely to belong to cohesive social networks, i.e., ones where the members have strong affinity for each other. (4) People with disabilities are less likely to be in social groups whose members have much in common. To help overcome these barriers, the author recommends that organizations provide structured socialization programs and formal mentors, as well as regular sensitivity training.

Perales (2022) investigated the factors associated with workplace well-being among LGBTQ employees. The author used secondary data from the 2020 Australian Workplace Equality Index (AWEI) Employee Survey, They found that LGBTQ employee workplace well-being was associated with having met or exceeded expectations regarding employee networks, ally training, and having visible allies in their organizations.

## 4 Discussion and future direction for research

### 4.1 ONA efficacy for workplace inclusion and belonging

Understanding the complexities and nuances of organizational networks is vital to fostering workplace inclusion and belonging. The majority of articles included in this review focused on gender-related outcomes, while others included outcomes related to race, ethnicity, and culture, academic medical centers, employees of a multinational organization, individuals with disabilities, and LGBTQ employees.

The scoping literature review demonstrated that ONAs can be a useful tool to better understand how people of different genders function differently within organizations, and to analyze the outcomes of initiatives aimed at improving gender inclusion in the workplace. Findings indicated that woman-to-woman networks are important for career growth and ONA can be used as a tool to inform related interventions (Hernandez et al., 2022); gender inclusivity on board of directors would likely have varying results that could be explored using ONA (de Mateos Cabo et al., 2021); and ONA can provide insights related to gender imbalances in hiring and promotion (Carboni et al., 2022). As an example, creation of an organizational network in which the highest echelons are populated by one gender and the other is more peripheral, less connected, or connected in homogenous clusters, would lead to the conclusion that there may be bias in the organization in need of remediation.

### 4.2 Differences in workplace networks

ONA tools have also been used to identify the impact of diversity on groups. For example, it has been determined that a lack of racial, ethnic and cultural differences among groups of employees can lead to a homophily effect where there is less collaboration between groups. On the other hand, network heterogeneity tends to lead to divergent viewpoints which can increase collaboration and network resiliency. ONA has also been used to identify that a key driver of perceived inclusion is a connection to a network of resources and access to well-connected site leaders, as a source for professional guidance (Hunt et al., 2009).

At their best, diverse networks are inherently indicative of multiple perspectives and innovative ideas. Ideally, member engagement results in additional connections and learning. Through cohesive connections, enhanced development leads to growth and increased success, both personally and professionally. However, often times due to unconscious bias, individuals tend to struggle to build diverse networks, and lack of contact between diverse groups tends to result in miscommunication and mistrust. Effective networks that are characterized by diversity tend to encourage trust and favor reciprocity. Thus, the intentionality of building organizational networks that lead to positive outcomes, then, is integral to success.

### 4.3 Future direction for research

The results of this review indicate that ONA tools, as they relate to inclusion and belonging in the workplace, have been most widely used for understanding and intervening on gender inequities that exist in the work setting and identifying the consequences of those inequities. Similar designs have been applied to a smaller number of studies with other underrepresented populations, such as racial and ethnic minorities, LGBTQ individuals and people with disabilities. Overall themes from the articles included: (1) the importance of utilizing organizational networks to understand the barriers more fully to workplace belonging and inclusion; (2) the benefits of meaningful connections within organizations, particularly for those who are traditionally underrepresented; and (3) the need to address limitations of organizational networks and break down barriers to career advancement for traditional underrepresented groups. Furthermore, ONAs reveals the importance of networks not only in the sphere of individual wellbeing but also at the organizational level. This finding aligns with current research that demonstrates diversity in organizations is directly correlated with organizational performance (Gomez and Bernet, 2019). Future studies are warranted that include: randomized controlled trials (RCT) designs, the analysis of men’s networks, the use non-binary definitions of gender, other traditionally underrepresented populations, as well as studies in healthcare settings.

### 4.4 Limitations

The studies included in this review were largely cohort, survey, and cross-sectional designs. No RCT were identified, which is considered the gold standard for identifying causal relationship between interventions and outcomes. Therefore, it would be advantageous for studies aimed at investigating the impact of ONA interventions to employ RCT designs to reduce bias and separate out whether other variables likely impacted the outcomes related to communication pattern changes and other ONA-related measures.

From a gender perspective, the papers included in this review largely focused on assessing women’s networks, and supporting women’s advancement in the work setting, indicating there is a dearth of evidence regarding the assessment and influence of men’s networks in the workplace, as well as those who identify as non-binary.

While there are a number of studies included in this review related to gender and race, ethnicity, culture outcomes, there are far fewer focused on other populations that tend to be underrepresented in the workplace, including veterans, LGBTQ individuals, people with disabilities, and older and younger employees. In addition, only two studies were conducted in an academic medical setting, despite the call to action for healthcare leaders and organizations to transform their practices in order to effect meaningful organizational change that leads to adaptability to external change and reduces silos that impeded innovation (Auton, 2014). As the authors in the call to action indicate, ONA can serve as a tool to “reduce and minimize points of overload” in networks and “build connectivity to individuals and functions that are on the network periphery” (Auton, 2014).

## 5 Conclusion

This scoping review demonstrates the usefulness of ONAs as a tool for better understanding and addressing barriers to workplace inclusion and belonging. While most studies included in this paper examined gender-based outcomes, findings across the literature highlighted an ONA’s ability to undercover often invisible barriers of exclusion based on gender, race, ethnicity and other demographic factors. The studies showed how ONAs can identify network structures that may disadvantage underrepresented groups, such as limited connectivity to key decision-makers or through the formation of homogenous clusters. Importantly, this scoping review reveals a need for future research employing different methodologies to establish causal relationships between ONA-informed interventions and workplace inclusion. Furthermore, future studies should expand beyond the predominantly gender-focused research to encompass a broader range of underrepresented populations. This expanded research will provide a more thorough understanding on how ONAs can be leveraged to enhance belonging in the workplace.
